# Supplementary figures and images for: Extract of Scutellaria baicalensis induces semaphorin 3A production in human epidermal keratinocytes
Source: PLoS One. 2021 Apr 27;16(4):e0250663. doi: 10.1371/journal.pone.0250663 (PMC8078742; doi:10.1371/journal.pone.0250663)

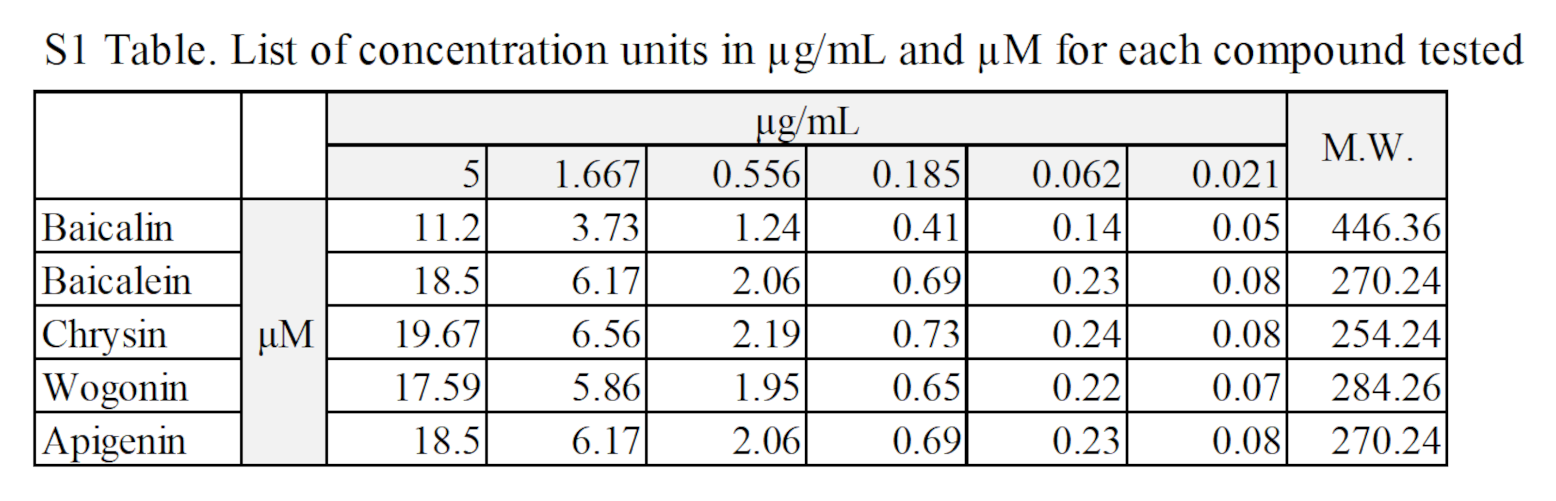

Supplement: S1 Table — (TIF) [file pone.0250663.s001.tif]
