# Supplementary material for: Extract of Scutellaria baicalensis induces semaphorin 3A production in human epidermal keratinocytes
Source: PLoS One. 2021 Apr 27;16(4):e0250663. doi: 10.1371/journal.pone.0250663 (PMC8078742; doi:10.1371/journal.pone.0250663)
Supplement: Attachment — Submitted filename: review for PONE-D-20-28160.pdf [file pone.0250663.s003.pdf]

PONE-D-20-28160

Extract of Scutellaria baicalensis induces semaphorin 3A production in human epidermal keratinocyte

Reviewer's comment

Authors are interested in an importance of epidermal Sema3A level to prevent sensory nerve fiber elongation leading to serious scratching in pruritic skin diseases such as AD and xerosis. This manuscript describes herbal plant extracts were tested for an *in vitro* Sema3A induction activity in normal human keratinocytes (NHEKs). The major findings were obtained from gene promoter assay by Sema3A promoter that authors characterized, qPCR of Sema3A mRNA, ELISA of secretory Sema3A protein, and human skin equivalent model. The authors showed results of the primary screening of gene promoter activity for 91 herbal plant extracts. In further screening by qPCR of mRNA, the remaining was five herbal extracts No.2, 7, 10, 13 and 76. Finally, S. baicalensis extract was selected as the best one in screened samples. In addition, baicalein and its glucuronide are proved to be main active component among 5 flavonoids included in S. baicalensis.

Although the data are interesting and authors are expected to progress this research in future, I have the following concerns regarding the experiment and interpretation of the data.

1. Page 2 line 22: "Previous studies" is "Our previous studies".

2. Page 3 line 36-39: Do authors think whether or not the active extract-induced Sema3A may lead to keratinocyte differentiation or migration?

3. Page 5 line 74: Authors mentioned Sema3A ointment in Abstract. Please add the reference here.

4. Page 6 line 87: Sample preparation is mentioned more in detail in the text.

5. Page 8 line 105, Page 11 line 160-161: According to pGL3-Sema3A vector containing luciferase gene, the sentence "Cultured NHEKs were……with luciferase reporter gene constructs containing -1444 bp of the human Sema3A promoter" is shown in Page 11, but more specified promoter sequence is shown in the text.

6. Page 10 line 143-146: Human skin equivalent model is supported by a little bit of more explanation for the readers.

7. Page 11 line 153: Statistical numbers (n) of the experiments are shown in the figures and legends.

8. Page 17 line 240-250: Did authors quantitate baicalein and other components in S. baicalensis extract that authors tested?

9. Page 20 line 296: It is referred that S. baicalensis contains 10 % baicalin and 1% baicalein. Which is the more active component? Do Authors guess β-glucuronidase degrades baicalin faster, and leads to bioactive component baicalein? If so, smooth β-glucuronidase degradation must be shown by an enzymatic experiment of culturing of keratinocyte or incubating of cell lysates.

10. Page 21 line 311-312: Authors reported that Sema3A mRNA expression in NHEKs is regulated by calcium. How is the possibility of activation due to contaminated calcium in the tested samples?

11. Fig. 5 and the legend: Chemicals have been compared at same molar concentration. Chemicals are shown in the figure at each of molar concentrations. In the legend, each name of chemicals is followed by both units of μg/mL and μM.
