# Supplementary material for: Extract of Scutellaria baicalensis induces semaphorin 3A production in human epidermal keratinocytes
Source: PLoS One. 2021 Apr 27;16(4):e0250663. doi: 10.1371/journal.pone.0250663 (PMC8078742; doi:10.1371/journal.pone.0250663)
Supplement: Attachment — Submitted filename: Review Comments.docx [file pone.0250663.s002.docx]

Some skin diseases such as atopic dermatitis (AD) and xerosis is mainly due to hyperinnervation in the epidermis. And Semaphorin 3A (Sema3A) is a nerve repulsion factor expressed in keratinocytes and it suppresses nerve fiber elongation in the epidermis. Therefore the paper of Yasuko and co-workers find that S.baicalensis extract and its compounds, baicalin and baicalein, may be promising candidates for improving histamine-resistant itch via the induction of Sema3A expression in epidermal keratinocytes. Thus, the study may provide a new therapeutic candidates for improving histamine-resistant itch caused by epidermal hyperinnervation.

As such, the matter is of interest, however the paper suffers for some limits:

1) In Fig 3, It is unconvincing that simply use ELISA to measure the Sema3A protein levels in the culture supernatants of NHEKs treated with five tested samples. Sema3A is not only the secreted protein, but also abundant in intracellular of NHEKs. It is recommended to use WB or immunofluorescence to observe the protein levels in different treatment groups.
2) In Fig 3, samples No. 2, and 13 can increase the expression levels of Sema3A in cultured NHEKs at 24 hours. Do these drugs have any effect on cell viability?
3) In Fig5a, the results showed that the luciferase activities in cultured NHEKs were increased dose dependently by the application of baicalin orbaicalein. Are there statistically significant differences as compared to the control ?

Once the above concerns are fully addressed, the manuscript could be accepted for publication in this journal.
