# Supplementary material for: Extract of Scutellaria baicalensis induces semaphorin 3A production in human epidermal keratinocytes
Source: PLoS One. 2021 Apr 27;16(4):e0250663. doi: 10.1371/journal.pone.0250663 (PMC8078742; doi:10.1371/journal.pone.0250663)
Supplement: Attachment — Submitted filename: Response to Reviewers.docx [file pone.0250663.s004.docx]

**Response to Academic Editor**

We have responded to your three requirements below.

**1. Please ensure that your manuscript meets PLOS ONE's style requirements, including those for file naming.**

**Reply**

Thank you for your suggestion. We have adjusted the style of the manuscript, including file naming, to accord with PLOS ONE style requirements.

**2. Amendment of Funding Statement and Competing Interests Statement**

**Reply**

Thank you for your suggestion. We have amended Funding Statement and Competing Interests Statement in our cover letter.

**3. We note that you have included the phrase “data not shown” in your manuscript. Unfortunately, this does not meet our data sharing requirements. PLOS does not permit references to inaccessible data.**

**Reply**

Thank you for your suggestion. We have deleted a sentence with the phrase "data not shown" in the Results section, as the data are not a core part of the research presented in this study.

•Results: Page 16, lines 206-207. The underlined sentence has been removed from the revised manuscript.

In samples No. 10 and 76, they were slightly increased at 3 and 6 hours, respectively. No enhancement of Sema3A mRNA expression was noted in any group at sample treatment later than 6 hours (data not shown).

**Response to Reviewer #1**

Reviewer #1: This is an interesting article on the development of a topical solution from plant extracts that utilizes small molecules that can penetrate the skin barrier to induce the production of the nerve repulsion factor Sema3A. The article is well written and the experimental protocol as well as the statistical analysis are sound.

**Reply**

Thank you for your supportive peer-review comments.

**Response to** **Reviewer #2**

Reviewer #2: Some skin diseases such as atopic dermatitis (AD) and xerosis is mainly due to hyperinnervation in the epidermis. And Semaphorin 3A (Sema3A) is a nerve repulsion factor expressed in keratinocytes and it suppresses nerve fiber elongation in the epidermis. Therefore the paper of Yasuko and co-workers find that *S. baicalensis* extract and its compounds, baicalin and baicalein, may be promising candidates for improving histamine-resistant itch via the induction of Sema3A expression in epidermal keratinocytes. Thus, the study may provide new therapeutic candidates for improving histamine-resistant itch caused by epidermal hyperinnervation. As such, the matter is of interest, however the paper suffers for some limits:

**1) In Fig 3, it is unconvincing that simply use ELISA to measure the Sema3A protein levels in the culture supernatants of NHEKs treated with five tested samples. Sema3A is not only the secreted protein, but also abundant in intracellular of NHEKs. It is recommended to use WB or immunofluorescence to observe the protein levels in different treatment groups.**

**Reply**

Thank you for these peer-review comments. In this revision, we checked the levels of Sema3A proteins in cell lysates from cultured NHEKs treated with the two test samples (No. 2 and 13) using ELISA. As with the culture supernatant, a significantly increased level of Sema3A was found in the cell lysate of cultured NHEKs treated with sample No. 13, but not with sample No. 2. Thus, it was concluded that only the extract of *S. baicalensis* had consistent Sema3A-inducing activity in cultured NHEKs at the gene expression and protein production levels. We added these data as Fig 3B and revised several sentences in the Abstract, Materials and Methods, Results, Discussion, and Figure legends accordingly.

•Abstract: Page 2, line 12-14. We revised the underlined sentence.

Among the positive samples, only the extract of *S. baicalensis* was found to consistently increase Sema3A levels in cultured NHEKs in assays using quantitative real-time PCR and ELISA.

•Materials and methods: Page 10, line 128-131. We added the underlined sentence.

Cells cultured for 6 or 24 hours were lysed in M-PER mammalian protein extraction reagent and protease inhibitor cocktail (Thermo Scientific, Waltham MA, USA), then Sema3A levels in the cell lysate were measured using a Human Sema3A ELISA kit (Elabscience, Houston, TX, USA).

•Results: Page 16, line 208-209. We revised the figure numbers from Fig 3 to Fig 3A with the addition of Fig 3B.

Sema3A protein levels were measured in the culture supernatants of NHEKs treated with five tested samples using ELISA (Fig 3A).

•Results: Page 17, line 215-217. We added the underlined sentence.

Increased expression at the protein level was also observed using ELISA with cell lysates of cultured NHEKs with sample No. 13 but not with sample No. 2 (Fig 3B).

•Figure Legends: Page 18, lines 235-238. We revised and added the underlined sentences.

(B) NHEKs were incubated with either sample No. 2 (*A. montana*) or No. 13 (*S. baicalensis*) at 50 μg/mL for 6 and 24 hours. After that, the cell lysates were prepared with lysis buffer, and then the levels of Sema3A protein in the cell lysates were measured using ELISA.

•Discussion: Page 21, lines 295-301; Page 22, line 302. We revised the underlined sentences.

In addition, the extract of *S. baicalensis* significantly increased the level of Sema3A protein in the supernatants of NHEKs cultured for 24 hours (Fig 3A) and in cell lysates from NHEKs cultured for 6 and 24 hours (Fig 3B). Meanwhile, no significant increase in the level of Sema3A protein in culture supernatants or cell lysates from NHEKs treated with an extract of *A. montana* were found in this study. These findings suggest that the extract of *S. baicalensis* consistently has Sema3A-inducing activity in cultured NHEKs on both gene and protein expression levels.

**2) In Fig 3, samples No. 2, and 13 can increase the expression levels of Sema3A in cultured NHEKs at 24 hours. Do these drugs have any effect on cell viability?**

**Reply**

Thank you for this suggestion. We tested the effects of samples No. 2 and No. 13 on cell viability of cultured NHEKs. In the cell counting assay, these plant extracts significantly increased cell viability at 6 hours of treatment, whereas cell viability decreased at 24 hours.

We added these data as Fig 3C and added and revised several sentences in the Materials and methods, Results, Figure legends, and Discussion.

•Materials and methods: Page 11, line 133-140. We added the underlined sentences.

Cell counting assay

A Cell Counting Kit (CCK)-8 (Dojindo, Kumamoto, Japan) was used to assess the cell toxicity of each compound on NHEKs. Cells were seeded at 1 × 10^4^ cells per well in 96-well culture plates. After incubation overnight at 37°C, an extract of *S. baicalensis* or *A. montana* (50 μg/mL) was added to each well, then incubated for 6 or 24 hours at 37°C. At each time point, CCK-8 solution was added to each well and incubated for 3 hours at 37°C. Cell viability was estimated by measuring the absorbance at 450 nm using a 2030 ARVO-X4 Multi label Plate Reader (Perkin Elmer).

•Results: Page 17, lines 217-221. We added the underlined sentences.

We further tested the effects of samples No. 2 and No. 13 on cell viability of cultured NHEKs using a cell counting assay. In the assay, the cell viabilities of NHEKs treated with these compounds was significantly increased at 6 hours compared with untreated controls, whereas samples No. 2 and No. 13 reduced cell viabilities to 89.8% and 78.2% at 24 hours, respectively (Fig 3C).

•Figure legend: Page 18, line 238-241. We added the following sentences.

(C) Cell viability of cultured NHEKs treated with an extract of *A. montana* or *S. baicalensis* was measured using a cell counting assay. All results are shown as mean ± S.D. of three independent experiments. ^*^ p < 0.05, ^**^ p < 0.01, ^***^ p < 0.001, ^****^ p < 0.0001 (two-way ANOVA followed by Dunnett’s test).

•Discussion: Page 21, line 295-301; Page 22, line 302-308. We added the underlined sentences.

In addition, our cell counting assay showed that extracts of *S. baicalensis* and *A. montana* significantly increased cell viability at 6 hours, whereas the cell viabilities with *S. baicalensis* and *A. montana* treatments at 24 hours reduced to 89.8% and 78.2%, respectively (Fig 3C), although no morphological findings related to cytotoxicity were observed in microscopic observations up to 24 hours. Thus, these findings raise the possibility that extracts of *S. baicalensis* and *A. montana* are at least partly involved in cell proliferation and/or differentiation rather than cytotoxic events.

**3) In Fig 5a, the results showed that the luciferase activities in cultured NHEKs were increased dose dependently by the application of baicalin or baicalein. Are there statistically significant differences as compared to the control?**

**Reply**

Thank you for this question. In this revision, we repeated the experiment and performed reproducibility and statistical processing of the results. We revised this data as a new Fig 5A and revised several sentences in the Abstract, Results, Figure legends, Discussion.

•Abstract: Page 2, line 16-18; Page 3, line 19. We revised the underlined sentence.

In addition, we investigated which components in the extract of *S. baicalensis* contributed to Sema3A induction and found that baicalin and baicalein markedly increased the relative luciferase activity, and that baicalein had higher induction activity than did baicalin.

•Results: Page 20, line 270-277. We revised and added the underlined sentences.

The extracts of *S. baicalensis*, baicalin, and baicalein all significantly increased the relative luciferase activity at a concentration of 5 μg/mL, and the increase in activity of these samples was dose dependent. Notably, baicalein markedly increased its activity at a lower concentration of 0.56 μg/mL compared with the extracts of *S. baicalensis* and baicalin. Such significant alterations were observed with chrysin and apigenin but were not dose dependent. Meanwhile, wogonin significantly decreased luciferase activity at a concentration of 5 μg/mL.

•Figure legends: Page 21, line 286-288. We revised the underlined sentence.

All results are expressed as mean ± S.D. of three independent experiments. ^*^ p < 0.05, ^**^ p < 0.01, ^***^ p < 0.001, ^****^ p < 0.0001 (one-way ANOVA followed by Dunnett’s test).

•Discussion: Page 23 line, 334-337; Page 24, line 338-340. We revised the underlined sentences.

Our reporter gene assay showed that relative luciferase activities in cultured NHEKs were significantly increased in a dose-dependent manner by the application of extracts of *S. baicalensis*, baicalin, and baicalein compared with a control (Fig 5A). Notably, baicalein showed significantly higher activity at a low concentration (0.56 μg/mL) among the major bioactive flavones in *S. baicalensis*, suggesting that it is the most active compound.

•Discussion: Page 24, line 344-346. We revised the underlined sentence.

Taken together, these findings suggest that baicalin and its aglycone, baicalein, are the main active components in *S. baicalensis* contributing to Sema3A induction in keratinocytes.

•Discussion: Page 24, line 347-349. We revised the underlined sentence.

Among the four flavone aglycones tested, baicalein showed significantly higher activity in a dose-dependent manner, but no other aglycones, wogonin, chrysin, and apigenin.

**Once the above concerns are fully addressed, the manuscript could be accepted for publication in this journal.**

**Reply**

Thank you very much for these review comments.

**Response to Reviewer #3**

**Reviewer #3: Authors are interested in an importance of epidermal Sema3A level to prevent sensory nerve fiber elongation leading to serious scratching in pruritic skin diseases such as AD and xerosis. This manuscript describes herbal plant extracts were tested for an in vitro Sema3A induction activity in normal human keratinocytes (NHEKs). The major findings were obtained from gene promoter assay by Sema3A promoter that authors characterized, qPCR of Sema3A mRNA, ELISA of secretory Sema3A protein, and human skin equivalent model. The authors showed results of the primary screening of gene promoter activity for 91 herbal plant extracts. In further screening by qPCR of mRNA, the remaining was five herbal extracts No.2, 7, 10, 13 and 76. Finally, *S. baicalensis* extract was selected as the best one in screened samples. In addition, baicalein and its glucuronide are proved to be main active component among 5 flavonoids included in *S. baicalensis.* Although the data are interesting and authors are expected to progress this research in future, I have the following concerns regarding the experiment and interpretation of the data.**

**Reply**

Thank you very much for your corrections. We will answer these points one by one.

**1. Page 2 line 22: “Previous studies” is “Our previous studies”.**

**Reply**

Thank you for this suggestion. We have revised the part as suggested (page 2, line 5-6).

**2. Page 3 line 36-39: Do authors think whether or not the active extract-induced Sema3A may lead to keratinocyte differentiation or migration?**

**Reply**

Thank you for your comment. A previous study showed that morphological differentiation and increased expression of keratins 1 and 10 (K1/K10) were found in baicalein-treated HaCaT cells, which is a cell line of keratinocytes, without affecting ROS generation, cytochrome c release, or apoptosis (Huang et al., Exp Dermatol. 2016 Aug;25(8):623-9). Thus, although this study did not examine such effects, at least baicalein may promote keratinocyte differentiation. We have added the above report as No.16 in the Reference section and added a sentence in the Discussion section.

•Discussion: Page 22, line 309-310. We added the underlined sentence.

This idea might also be supported by a previous study that baicalein, one of the components in *S. baicalensi*s, induced keratinocyte differentiation [16].

**3. Page 5 line 74: Authors mentioned Sema3A ointment in Abstract. Please add the reference here.**

**Reply**

Thank you for this correction. We added an appropriate reference in this revision (page 5, line 58).

**4. Page 6 line 87: Sample preparation is mentioned more in detail in the text.**

**Reply**

Thank you for your comment. The method for sample preparation was revised in the Materials and methods section.

•Materials and methods: Page 6, line 73-78. We added and revised the underlined words.

Ethanol extracts of herbal plants were obtained from Maruzen Pharmaceuticals Co., Ltd. (Hiroshima, Japan) and Koei Kogyo Co., Ltd. (Tokyo, Japan). These ethanol extracts were evaporated under reduced pressure and lyophilized as test samples. Lyophilized samples were reconstituted at 100 mg/mL with DMSO, and each test sample was diluted with basal medium at a concentration in the range of 0.02–500 μg/mL and used in each experiment.

**5. Page 8 line 105, Page 11 line 160-161: According to pGL3-Sema3A vector containing luciferase gene, the sentence “Cultured NHEKs were……with luciferase reporter gene constructs containing -1444 bp of the human Sema3A promoter” is shown in Page 11, but more specified promoter sequence is shown in the text.**

**Reply**

Thank you for your comment. However, we are currently analyzing transcriptional regulators that bind to this promoter region, so we cannot show the Sema3A promoter sequence.

**6. Page 10 line 143-146: Human skin equivalent model is supported by a little bit of more explanation for the readers.**

**Reply**

Thank you for your suggestion. We added more detailed information about this. We have added and revised several sentences in the Materials and Methods, Results, and Figure title.

•Materials and methods: Page 11, line 142–148; Page 12, line 149–153. We have added and revised the underlined title and sentences.

Reconstructed human epidermal (RHE) models

RHE model (EPISKIN, SA, Lyon, France) is an *in vitro* reconstructed human epidermis from normal human keratinocytes cultured on an inert polycarbonate filter at the air-liquid interface, in a chemically defined medium, featuring normal ultrastructure and functionality similar to human tissue *in vivo* [15]. RHE models were cultured in accordance with the manufacturer’s protocol. Briefly, inserts containing RHE models were shipped at room temperature in a multi-well plate filled with an agarose-nutrient solution in which they were embedded. The inserts were carefully taken out of the multi-well plate, any remaining agarose adhering to the outer sides of the insert was removed, and placed in a plate in which each well had been filled with Maintenance Medium (EPISKIN, Lyon, France) overnight at 37°C, 5% CO_2_.

•Results: Page 18, line 243-246. We have revised the underlined title and sentence.

Effect of the plant extracts on Sema3A protein production in RHE models

The Sema3A-inducing activity of the extracts of *S. baicalensis* and *A. montana* was evaluated using RHE models.

•Figure title: Page 19, line 255-256. We have revised the underlined title.

Fig 4. Effects of *S. baicalensis* and *A. montana* extracts on Sema3A production in RHE models.

**7. Page 11 line 153: Statistical numbers (n) of the experiments are shown in the figures and legends.**

**Reply**

Thank you for this suggestion. The statistical numbers of the experiments were added in each figure legend of this revision.

•Table legends: Page 15, line 187. We have revised the underlined words.

All results are expressed as mean ± S.D. of triplicate samples.

•Figure legend (Fig 1): Page 16, line 197. We have revised the underlined words.

All results are expressed as mean ± S.D. of three independent experiments.

•Figure legend (Fig 2): Page 17, line 227-228. We have revised the underlined words.

All results are expressed as mean ± S.D. of three independent experiments.

•Figure legend (Fig 3): Page 18, line 239-240. We have revised the underlined words.

All results are expressed as mean ± S.D. of three independent experiments.

•Figure legend (Fig 4): Page 19, lines 260-261. We have revised the underlined words.

All results are expressed as mean ± S.D. of three independent experiments.

•Figure legend (Fig 5): Page 21, line 286-288. We have added the underlined words.

All results are expressed as mean ± S.D. of three independent experiments.

**8. Page 17 line 240-250: Did authors quantitate baicalein and other components in** **S. baicalensis extract that authors tested?**

**Reply**

Thank you for your suggestion. We did not accurately quantify the components contained in an extract of *S. baicalensis*. In another experiment in which an extract of *S. baicalensis* was fractionated using HPLC, we roughly estimated that baicalin and baicalein are contained in *S. baicalensis* extract in similar amounts as previous reports. We have revised a sentence more clearly in the Discussion section.

•Discussion: Page 24, line 342-344. We have revised the underlined sentence.

It has been previously reported that an extract of *S. baicalensis* contains approximately 10% baicalin and 1% baicalein [25-27], so baicalin is a major component of *S. baicalensis* extract.

**9. Page 20 line 296: It is referred that S. baicalensis contains 10 % baicalin and 1% baicalein. Which is the more active component? Do Authors guess beta-glucuronidase degrades baicalin faster, and leads to bioactive component baicalein? If so, smooth beta-glucuronidase degradation must be shown by an enzymatic experiment of culturing of keratinocyte or incubating of cell lysates.**

**Reply**

Thank you for your suggestion. We examined the effect of β-glucuronidase inhibitor on relative luciferase activity for Sema3A in baicalin-treated NHEKs. However, in the assay, β-glucuronidase inhibitor did not reduce the increase in relative luciferase activity for Sema3A in the baicalin-treated NHEKs. Subsequent literature research led to the idea that there are several types of β-glucuronidase ([Beta Glucuronidase - an overview | ScienceDirect Topics](https://www.sciencedirect.com/topics/pharmacology-toxicology-and-pharmaceutical-science/beta-glucuronidase)) in bacteria, humans, plants, etc. and that the bacterial β-glucuronidase converts baicalin to baicalein, but the glucuronidase present in human skin does not act on baicalin. Thus, we deleted the following sentences:

It has also been demonstrated that human skin contains substantial amounts of β-glucuronidase, and its activity is higher in the epidermis than in the dermis [27, 28]. Thus, because baicalin is hydrolyzed to baicalein and glucuronic acid by β-glucuronidase, the substantial induction of Sema3A expression in *S. baicalensis*-treated NHEKs may be brought about by the action of baicalein.

And we revised the sentence in the Discussion section.

•Discussion: Page 24, line 340-342. We have revised the underlined sentence.

Baicalein is a glucuronide conjugate (Fig 5B) and is known to be converted to its aglycone, baicalein, by β-glucuronidase derived from bacteria but not that from humans [24].

**10. Page 21 line 311-312: Authors reported that Sema3A mRNA expression in NHEKs is regulated by calcium. How is the possibility of activation due to contaminated calcium in the tested samples?**

**Reply**

The purchased baicalin and baicalein did not contain calcium, and then when they were lysed and used in the promoter assay, the promoter activity of Sema3A increased in NHEKs treated with them as shown in the Fig 5A. Therefore, although the effect of calcium in crude extracts cannot be completely eliminated, it is suggested that the effect of calcium on the increase in promoter activity and expression of Sema3A in the samples tested in this study was considered to be low.

**11. Fig. 5 and the legend: Chemicals have been compared at same molar concentration. Chemicals are shown in the figure at** **each of molar concentrations. In the legend, each name of chemicals is followed by** **both units of ug/mL and uM.**

**Reply**

Thank you for the concern. As pointed out, we have added a list of both units of µg/mL and µM for each compound as S1 Table, and we revised sentences in the Results and Supporting information section.

•Results: Page 20, line 268-270. We added the underlined sentences.

The experiment was performed with a maximum concentration of 5 μg/mL and a 3-fold dilution series. The molar concentration of each compound is shown in S1 Table.

•Supporting information: Page 31, line 468-469. We have added the Supporting information section and the table titles as follows.

Supporting information

S1 Table. List of concentration units in µg/mL and µM for each compound tested
